# MemDis: Predicting Disordered Regions in Transmembrane Proteins

**DOI:** 10.3390/ijms222212270

**Published:** 2021-11-12

**Authors:** Laszlo Dobson, Gábor E. Tusnády

**Affiliations:** Institute of Enzymology, Research Centre for Natural Sciences, Magyar Tudósok Körútja 2, 1117 Budapest, Hungary; dobson.laszlo@ttk.hu

**Keywords:** transmembrane proteins, intrinsically disordered proteins, deep learning, convolutional neural network, bidirectional long-short term memory

## Abstract

Transmembrane proteins (TMPs) play important roles in cells, ranging from transport processes and cell adhesion to communication. Many of these functions are mediated by intrinsically disordered regions (IDRs), flexible protein segments without a well-defined structure. Although a variety of prediction methods are available for predicting IDRs, their accuracy is very limited on TMPs due to their special physico-chemical properties. We prepared a dataset containing membrane proteins exclusively, using X-ray crystallography data. MemDis is a novel prediction method, utilizing convolutional neural network and long short-term memory networks for predicting disordered regions in TMPs. In addition to attributes commonly used in IDR predictors, we defined several TMP specific features to enhance the accuracy of our method further. MemDis achieved the highest prediction accuracy on TMP-specific dataset among other popular IDR prediction methods.

## 1. Introduction

Transmembrane proteins (TMPs) are located in different membranes and they provide gates between the inner and outer side of cells or organelles. Around 25% of the coded proteins in the human proteome contain one or more membrane regions [1]. These segments embedded in the lipid bilayer are structurally well defined; however, their tail and loop regions often contain unstructured segments. Such regions are aiding various functions from providing flexible linkers to binding motifs for other molecules [2]. Although intrinsically disordered regions (IDRs) are well studied in general, the currently available prediction methods have limited accuracy on membrane proteins for several reasons [3]. On the one hand, protein disorder is conditional [4] and heavily influenced by the environment; thus, membrane proteins, exposed on both outside and inside spaces, cannot be well described using a single function or machine learning algorithm. Moreover, lipid components of the membrane influence the charge and acidity near the transmembrane regions, further complicating the situation. On the other hand, these methods are generally trained on mixed protein sets predominantly containing non-TMPs, resulting in biased information from the perspective of TMPs. Here, we propose MemDis, a novel tool for predicting IDR regions in TMP proteins, which achieved the highest accuracy among tested methods. We utilized Convolutional Neural Networks (CNNs) to capture local features of the sequence represented by Position-Specific Scoring Matrix and Long Short-Term Memory (LSTM) Network to take advantage of the semantic properties of the protein sequence.

## 2. Results

To realistically capture the different flavors of disorder in membrane proteins, four different models were created according to different topological regions. CNNs were trained on extracellular-distant (distance from membrane >15aa), proximal- (≤15aa) and intracellular-distant (distance >15aa), proximal (≤15aa) residues separately. A bidirectional LSTM network was also trained to “smooth” the prediction of CNNs on individual residues and achieve better sensitivity.

**Figure 1:**
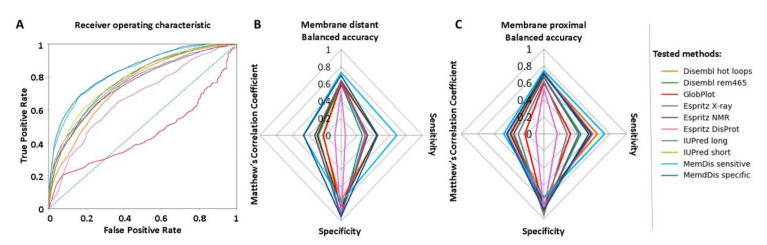
(**A**) Receiver operating characteristic of MemDis and other disorder prediction methods. (**B**) Averaged performance of membrane-distant predictors. (**C**) Average performance of membrane proximal predictors.

Based on the training and validation set, we found that the CNNs, with a slightly higher cutoff (0.65—notably this result is scaled so the web server will display 0.5 cut-off) and a ±4 residue smoothing achieved the best specificity, while also keeping other metric values considerably high. In contrast, the LSTM with a ±7 residue smoothing had the best sensitivity. Both versions (from now on referred to as specific and sensitive, respectively) achieved a remarkable 0.83–0.84 Area Under Curve (AUC) (Figure 1A, Supplementary Materials). We compared the results of our method to other popular algorithms [5,6,7,8] using metrics from the most recent CAID experiment [9] (Supplementary Table S1). We used the complete protein sequence for testing; however, we only considered fragments selected earlier for the evaluation. Some of the tested methods achieved slightly better specificity, at the cost of barely predicting disordered segments. The best sensitivity was achieved using the MemDis sensitive. Although dozens of IDR prediction methods are available, when selecting other methods, we aimed to select ones with slightly different methodology (machine learning, biophysical approaches) and training sets (X-ray, NMR, etc.). Both the sensitive and specific settings of MemDis achieved the highest balanced accuracy, Matthew’s Correlation Coefficient (MCC) and AUC (Figure 1A, Supplementary Materials). Notably, MemDis uses different models to predict membrane-distant and proximal regions, and their separate performance also captures disorder better compared to other methods (Figure 1B,C; Supplementary Table S1, Supplementary Materials). When evaluating IUPred3 locally, experimental filtering was not used.

MemDis is available on GitHub at https://github.com/brgenzim/MemDis. Since the local installation is slightly complicated as users have to set up all dependencies as well, we also prepared a webserver (available at http://memdis.ttk.hu), where users can query their sequence(s). The webserver displays topology predicted by CCTOP and a graph for disordered prediction.

**Figure 2:**
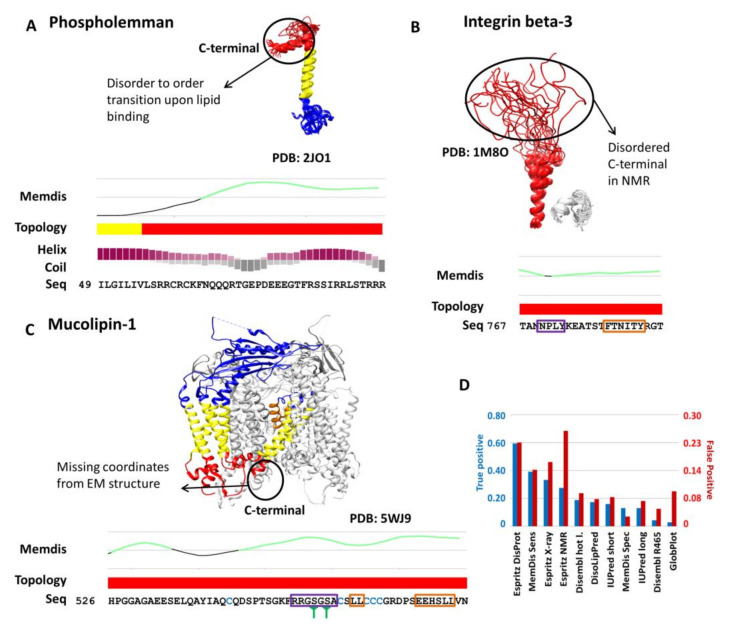
Interpretation of MemDis results. (**A**) Phospholemman: solution NMR structure, and representation of C-terminal by the prediction of MemDis, CCTOP and FELLS (helical propensity: purple, coil propensity: grey). (**B**) Integrin beta-3: solution NMR structure, MemDis and CCTOP predictions. The proposed NPxY endocytosis sorting signal is marked with purple, the LIR autophagy motif is marked with an orange box. (**C**) Mucopilin-1: Electron-microscopy structure, prediction from MemDis and CCTOP. Phosphoserines are marked with green cones below the sequence. The phosphorylation site is marked with a purple box, di-leucine motifs are marked with orange boxes. Cysteines have blue color. Topology is represented both in the structures and topology lines and structures are colored blue, red, yellow and orange (extracellular, cytosolic, transmembrane, and re-entrant loop regions, respectively). Disordered regions from MemDis are marked with green lines on the graphs. Note, only specific regions of the sequences are shown. (**D**) Detection rate of lipid-binding and non-lipid-binding disordered regions from the MemMoRF database.

We also checked a handful of well-defined examples where the output of MemDis is supported by literature evidence. Phospholemman is a member of the FXYD family that regulates ion transport [10]. The cytosolic C-terminal tail was shown to associate with the micelle surface [11], forming a helical structure upon binding. MemDis predicts this region as disordered. The helical propensity prediction of FELLS [12] suggests that this region is likely helical (Figure 2A). Thus, combining the MemDis and other secondary structure prediction methods, lipid binding can be assumed for membrane proximal regions. Integrin alpha-IIIb is a receptor protein with a cytosolic disordered tail according to DisProt [13], exhibiting short linear motifs (SLiMs) proposed to play a role in SARS-CoV-2 infection [14]. Membrane proximal disordered regions are often missed by prediction methods, making it hard to find novel linear motif candidates; however, MemDis successfully detects these regions (Figure 2B). Mucolipin-1 is a cation channel, probably playing a role in membrane trafficking. The C-terminal cytosolic region has five cysteines, a residue that is often referred to as order-promoting (as they can form disulphide bridges in an extracellular environment), which deceives many predictors. MemDis has a built-in topology filter and predicts this region as disordered, in agreement with the electron-microscopy structure lacking coordinates for this region [15]. The C-terminal cytosolic tail of Mucolipin is also stacked with SLiMs: it has two di-leucine motifs [16], and phosphoserines [17] in the well-defined PKA phosphorylation site [18], further supporting that the C-terminal is disordered (Figure 2C).

We also assessed how predictors work to predict lipid-binding regions. MemMoRF is a novel database of disordered regions that undergo disorder-to-order transition upon membrane binding [19]. We measured the accuracy of different prediction methods on such regions. Unfortunately, all methods have poor performance (−0.19–0.03 MCC, Supplementary Table S1) on this dataset when measuring residue level accuracy. To overcome this, we counted the number of regions that have at least 60% of their residues predicted as disordered. In this comparison, Espritz DisProt had the highest hit rate, however, on the price of predicting many false positive regions too, while MemDis with sensitive settings was second, with somewhat fewer false positive regions (Figure 2D). We also evaluated DisoLipPred [20], which was developed specifically to find lipid-binding regions; however, it detected only 20% of lipid-binding disordered regions. In sum, none of the methods are capable of detecting such information reliably alone; however, introducing additional filters (topology, secondary structure) may increase their accuracy, as it was shown on MemDis in the case of Phospholemman.

## 3. Materials and Methods

**Figure 3:**
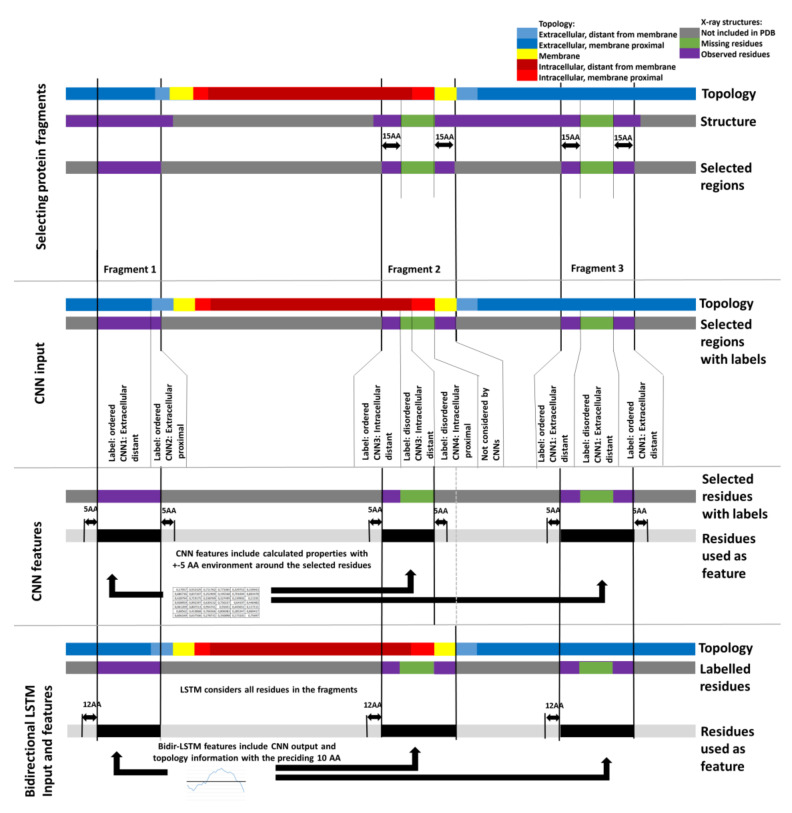
Data preparation for the training of MemDis. First, we selected protein fragments based on the available PDB information. Extracellular-distant (distance from membrane >15 AA), proximal (<15AA) and intracellular-distant, proximal residues from these fragments were fed into the appropriate CNN, also considering information from residues within 5AA from the residue of interest. The LSTM was trained on the full-length protein fragments considering the preceding 10AA.

We downloaded the MobiDB database [21] in 1April 2021, and selected the missing residues (th_90, used as disordered label) and observed (th_90, used as ordered label) subsets, defining regions from X-ray structures when there is 90% agreement between the observations. Next, we used CCTOP [22] to filter TMPs and used CD-HIT [23] to reduce redundancy to 40% sequence identity (Supplementary Table S2). In most cases, the full protein structure was not solved, so we used fragments of the protein sequences. First, we selected every IDR together with flanking ordered regions up to 15aa if they were included in the PDB. Next, we randomly selected ordered regions (Figure 3). The fragments were randomly selected into the train, validation and independent test set (Supplementary Table S3). We prepared Convolutional Neural Networks (CNNs) and a bidirectional Long Short-Term Memory (LSTM) network to predict IDRs.

For the CNNs, each non-membrane residue in this dataset belonged to one of the following four TMP topology categories: extracellular-distant (distance from membrane >15aa), proximal (≤15aa) and intracellular-distant (distance >15aa), proximal (≤15aa). Disordered and ordered residues were selected in a way that their distributions be roughly equal in each topological subset (max. 10% difference, Supplementary Table S4). We prepared four convolutional neural networks (CNNs) for the four topological regions (Figure 3). The features (Supplementary Table S5) include amino acid distribution, non-redundant AAIndex [24] categories (i.e., different amino acid scales), ProtParam [25] features (i.e., molecular weight, isoelectric point and instability index), topology information based on CCTOP and PSI-BLAST results. We also used Netsurfp [26] to predict accessibility of residues and SEG implemented in PlatoLoco [27] to detect low complexity regions. We used a ±5 length window around each residue and calculated 39 features for them, this way producing a feature matrix of size 11 × 39 (Supplementary Table S5) that was fed into the appropriate CNN (this window may contain residues not included in PDB or transmembrane residues, as these residues are only used as features belonging to a properly labelled residue). The CNNs were trained until their validation loss stopped decreasing for a constitutive 10 epochs (this occurred roughly at 1000 epochs)—the training and the validation accuracy at this point did not show high differences (Supplementary Table S6).

The bidirectional Long Short-Term Memory (LSTM) was trained on the full length fragments (including membrane regions) and used the output of the CNNs with topology information to predict disordered regions. Since the CNNs can only predict residues in an aqueous environment, for membrane residues the LSTM received “0” value as input. The LSTM was set to consider the preceding 12 time steps (Figure 3). The parameters of the CNNs and LSTM are available in Supplementary Table S7.

For testing, we hold back each hit from PSI-BLAST that occurred during training to avoid data leakage. Since the redundancy filter was originally performed on full-length proteins, we ensured again that no fragment in the independent testing set shared 40% or higher sequence identity to any sequence in the training and validation sequence fragment sets.

To define lipid-binding regions, we used the MemMoRF [19] database. We used redundancy filtering to 40%, and excluded proteins from the training set of MemDis. The negative set was generated using fragments near to the membrane (15AA), that did not have lipid-binding annotation in MemMorRF.

## Data Availability

MemDis is available online at http://memdis.ttk.hu and for local use it can be downloaded from https://github.com/brgenzim/MemDis.

## Supplementary Materials

The following are available online at https://www.mdpi.com/article/10.3390/ijms222212270/s1.
