# Supplementary material for: MemDis: Predicting Disordered Regions in Transmembrane Proteins
Source: Int J Mol Sci. 2021 Nov 12;22(22):12270. doi: 10.3390/ijms222212270 (PMC8623522; doi:10.3390/ijms222212270)
Supplement: Supplementary file 1 [file ijms-22-12270-s001.zip › SupplementaryMaterial.pdf]

MemDis: Predicting disordered regions in transmembrane proteins

Laszlo Dobson [1], Gábor E. Tusnády [1,*]

Supplementary Material

Finding the best cut-off for the prediction:

Neural Networks produce a real number for their output (between 0 and 1 in this case), often interpreted as a probability of the prediction. For MemDis we used Tensorflow to train our network, which uses accuracy to fit the model. This means, that the network will produce a real number as output, and using a 0.5 cut-off we can discriminate disordered and ordered residues. This cut-off will work best if we evaluate the method for accuracy, however in real scenarios sensitive (recognizing most disordered residues on the cost of some error), or specific (not recognizing all disordered residues, however detected ones are truly disordered) settings are desirable. Therefore we used the training set, and tried many different cut-offs to select the one that has the best performance considering sensitivity and specificity (but not for accuracy). We also smoothed the result, using a sliding window over the sequence, that averages neighbouring values.

Evaluation metrics:

First we construct a confusion matrix by comparing the predicted and the observed values:

Observed/Predicted	Positive	Negative
Positive	True positive	False negative
Negative	False positive	True negative

Then we can calculate the different metrics:

1. Sensitivity (True positive rate), the proportion of predicted positive results and all positive results (the predictor's ability to find disordered residues correctly):

$$Sn = \frac{TP}{TP + FN}$$

2. Specificity, the proportion of predicted negative results and all negative results (the predictor's ability to exclude ordered residues correctly):

$$Sp = \frac{TN}{TN + FP}$$

False positive rate can be derived from specificity (FPR=1-Sp)

3. Balanced Accuracy (ACC), the arithmetic mean of sensitivity and specificity, or the average accuracy obtained on either class:

$$ACC = \frac{Sn + Sp}{2} = \frac{\frac{TP}{TP + FN} + \frac{TN}{TN + FP}}{2}$$

4. Matthew's Correlation Coefficient, the balanced measures between two variables.

$$MCC = \frac{TP * TN - FP * FN}{\sqrt{(TP + FP) * (TP + FN) * (TN + FP) * (TN + FN)}}$$

5. F1 Score, the harmonic mean of the precision and recall

$$\mathrm{F1} = \frac{2 * \mathrm{TP}}{2 * \mathrm{TP} + \mathrm{FN} + \mathrm{FP}}$$

6. Positive prediction value, the proportion of positive results that are actually positive.

$$\mathrm{PPV} = \frac{\mathrm{TP}}{\mathrm{TP} + \mathrm{FP}}$$

7. Area Under the Curve (AUC)

How well a predictor separates positive and negative examples. TPR and FPR were plotted against each other at different probability cut-offs, generating a curve. Then area under the curve was calculated.

8. Segments Overlap (SOL), the comparison of the overlapping segments, where the overlapping regions length is taken into consideration. The value is between 0 and 1.:

$$SOL = \frac{1}{N} \sum_{S} \frac{\min OV(s1, s2) + \delta(s1, s2)}{\max OV(s1, s2)}$$

$$\delta(s1, s2) = \min \begin{cases} \max OV(s1, s2) - \min OV(s1, s2) \\ \min OV(s1, s2) \\ \mathrm{int}\left(\frac{\overline{s1}}{2}\right) \\ \mathrm{int}\left(\frac{\overline{s2}}{2}\right) \end{cases}$$

where N is the number of observed disordered residue, S is the number of segment pairs, s1 and s2 are the observed and predicted disordered region, minOV(s1,s2) is the overlap length, maxOV(s1,s2) is the extended overlap length (for which either of segment s1 or s2 has a disordered residue).
